# Environmental and sociocultural factors are associated with pain-related brain structure among diverse individuals with chronic musculoskeletal pain: intersectional considerations

**DOI:** 10.1038/s41598-024-58120-9

**Published:** 2024-04-02

**Authors:** Lisa H. Domenico, Jared J. Tanner, Angela M. Mickle, Ellen L. Terry, Cynthia Garvan, Song Lai, Hrishikesh Deshpande, Roland Staud, David Redden, Catherine C. Price, Burel R. Goodin, Roger B. Fillingim, Kimberly T. Sibille

**Affiliations:** 1 Department of Biobehavioral Nursing Science, College of Nursing, University of Florida, 1225 Center Drive, Gainesville, FL 32610 USA; 2 Department of Clinical and Health Psychology, University of Florida, 1225 Center Drive, Gainesville, FL 32603 USA; 3 Pain Research and Intervention Center of Excellence, University of Florida, 2004 Mowry Road, Gainesville, FL 32610 USA; 4 Department of Physical Medicine and Rehabilitation, University of Florida, 3450 Hull Road, Gainesville, FL 32607 USA; 5 Department of Anesthesiology, Division of Pain Medicine, University of Florida, 1600 SW Archer Road, Gainesville, FL 32610 USA; 6 Department of Radiation Oncology, University of Florida, 2000 SW Archer Road, Gainesville, FL 32610 USA; 7 Department of Radiology, University of Alabama at Birmingham, 619 19th Street South, Birmingham, AL 35294 USA; 8 Department of Rheumatology, College of Medicine, University of Florida, 1600 SW Archer Rd, Gainesville, FL 32610 USA; 9 Department of Biostatistics, University of Alabama at Birmingham, 1665 University Blvd #327, Birmingham, AL 35294 USA; 10 Department of Psychology, University of Alabama at Birmingham, Campbell Hall 415, 1300 University Blvd, Birmingham, AL 35223 USA; 11 Department of Anesthesiology, Washington University, 660 S Euclid Ave, St. Louis, MO 63110 USA; 12 Department of Community Dentistry and Behavioral Science, University of Florida College of Dentistry, 1329 SW 16th Street, Gainesville, FL 32610-3628 USA

**Keywords:** Environmental, Sociocultural, Ethnicity-race, Musculoskeletal pain, Neuroimaging, Neuroscience, Risk factors

## Abstract

Chronic musculoskeletal pain including knee osteoarthritis (OA) is a leading cause of disability worldwide. Previous research indicates ethnic-race groups differ in the pain and functional limitations experienced with knee OA. However, when socioenvironmental factors are included in analyses, group differences in pain and function wane. Pain-related brain structures are another area where ethnic-race group differences have been observed. Environmental and sociocultural factors e.g., income, education, experiences of discrimination, and social support influence brain structures. We investigate if environmental and sociocultural factors reduce previously observed ethnic-race group differences in pain-related brain structures. Data were analyzed from 147 self-identified non-Hispanic black (NHB) and non-Hispanic white (NHW), middle and older aged adults with knee pain in the past month. Information collected included health and pain history, environmental and sociocultural resources, and brain imaging. The NHB adults were younger and reported lower income and education compared to their NHW peers. In hierarchical multiple regression models, sociocultural and environmental factors explained 6–37% of the variance in pain-related brain regions. Self-identified ethnicity-race provided an additional 4–13% of explanatory value in the amygdala, hippocampus, insula, bilateral primary somatosensory cortex, and thalamus. In the rostral/caudal anterior cingulate and dorsolateral prefrontal cortex, self-identified ethnicity-race was not a predictor after accounting for environmental, sociocultural, and demographic factors. Findings help to disentangle and identify some of the factors contributing to ethnic-race group disparities in pain-related brain structures. Numerous arrays of environmental and sociocultural factors remain to be investigated. Further, the differing sociodemographic representation of our NHB and NHW participants highlights the role for intersectional considerations in future research.

## Introduction

The Global Burden of Disease indicates that over 1.71 billion people have chronic musculoskeletal (MSK) conditions worldwide, with osteoarthritis (OA) affecting over 343 million individuals^1^. Knee OA is a leading cause of physical disability and contributes to mental health decline, reduced work productivity, and loss of participation in life activities^2–4^. Despite an increased focus from the scientific and healthcare communities and recent advances in diagnostic imaging and therapeutic intervention, knee OA remains a significant impediment to health and well-being, with a predicted increase due to the growing number of aging individuals^5^.

A significant amount of research shows ethnic-race groups differ in the burden experienced from knee OA. A collective body of findings suggest that Non-Hispanic black (NHB) individuals with knee OA report higher pain severity^6–13^, greater degree of disability^8,14–22^, and decreased sense of control over pain^15,23,24^ compared to their non-Hispanic white (NHW) peers suffering from the same condition. However, the relationship between ethnicity-race and pain is more complex. Imbalances in the representation by different ethnic-race groups from different environmental and sociocultural backgrounds have likely contributed to observed disparities^25^. We and others have shown that with consideration for socioenvironmental factors such as education, income, neighborhood housing status, and experiences of discrimination, previously reported ethnic-race group disparities wane in clinical and experimental pain^26^.

Structural differences in brain regions involved in pain processing are associated with chronic pain. These differences have been observed in the rostral and caudal anterior cingulate cortices (rACC, cACC), amygdala, hippocampus, insula, medial prefrontal cortex (MPFC), dorsolateral prefrontal cortex (DLPFC), thalamus and the bilateral primary somatosensory cortex (S1)^27–31^. The relationships between environmental and sociocultural factors and brain structures are being addressed across disciplines with fewer studies in the context of chronic pain^32–35^. We have reported that socioenvironmental factors account for previously reported ethnic-race group differences in the temporal lobe brain regions, an area of the brain associated with greater dementia risk^36^. In fact, we found an added relationship was demonstrated such that increasing socioenvironmental risk and greater pain stage were associated with thinner temporal lobe cortices^36^. Identifying the environmental and sociocultural factors contributing to disparities in pain-related brain structures is a critical step toward determining modifiable targets and improving health for all^2,37^.

The National Institutes on Aging (NIA) Health Disparities Research Framework was developed to evaluate existing evidence and identify areas to address moving forward. The NIA Framework provides an organizational structure across four levels of analysis (environmental, sociocultural, behavioral, and biological) with a list of associated factors for each level^2,38^. The National Institute on Minority Health and Health Disparities (NIMHD) Research Framework expanded on the NIA Framework by also including levels of influence (individual, interpersonal, community, and societal) and domains of influence (similar to the NIA Framework level of analysis with the addition of a structural designation for environmental and healthcare system is an additional category)^39^. Guided by the NIA and NIMHD Health Disparities Research Frameworks, factors available in the dataset were used to assess environmental and sociocultural levels of analysis.

The current study aims to investigate the environmental and sociocultural contributions to pain-related brain regions among a diverse sample of adults with knee pain consistent with or at risk for knee OA. Regions of interest (ROIs) within the brain were selected based on findings from our previous work^30,40–42^ and the existing literature^27–29,31^. Areas investigated include the rostral and caudal anterior cingulate cortices (ACC), insula, medial prefrontal cortex (MPFC), bilateral primary somatosensory cortex (S1), dorsolateral prefrontal cortex (DLPFC), thalamus, amygdala, and hippocampus. We hypothesized that environmental and sociocultural factors will explain a statistically significant proportion of the variance in pain-related brain regions.

## Method

### Study population

The study is a cross-sectional analysis of participants recruited for the Understanding Pain and Limitations in Osteoarthritic Disease-2 study (UPLOAD-2). Community-dwelling adults between ages 45–85 years old who self-identified as NHB or NHW and presented with unilateral or bilateral knee pain in the past month from the University of Florida (UF) and the University of Alabama at Birmingham (UAB). Participants were recruited via multiple advertisement methods and clinic-based methods, as previously reported between August 2015 and May 2017^43^. Exclusion criteria for the UPLOAD-2 study included: (1) cognitive impairment; (2) use of opioids on a daily basis; (3) hospitalization for a psychiatric illness in the preceding year; (4) a history of acute myocardial infarction, heart failure or uncontrolled hypertension (BP > 150/95 mm Hg); (5) prosthetic knee replacements or other clinically significant surgery to the affected knee; (6) peripheral neuropathy; and/or (7) systemic diseases including rheumatoid arthritis, systemic lupus erythematosus or fibromyalgia. For the current investigation, the sample was determined by individuals with brain imaging data. This study follows the STROBE guidelines for reporting studies^44^.

### Procedures

Participants completed three study session visits which included a baseline health assessment, quantitative sensory testing, and brain imaging. All three sessions were completed within approximately one week between each session. Anthropometric measurements were obtained including waist circumference. Information acquired included participants’ health history, pain and function history, and sociocultural and psychosocial factors. The measures described are limited to those relevant to the current investigation.

### Measures

#### Clinical pain and disability

*Total Pain Sites (n* = *147).* Participants were asked if they had pain on more days than not over the past 3 months at bilateral sites across the body (0–28 sites). Pain sites served as a covariate for global pain severity in the model since the other pain measures were limited to the knee^45^. Increasing number of pain sites has been linked to worse health outcomes and three or more pain sites are considered widespread pain^46,47^.

#### Brain imaging

##### MRI acquisition

Individuals who completed a brain MRI were included in this cross-sectional analysis. Both sites (UAB and UF) acquired MRI data using a 3 Tesla Philips Achieva scanner (32-channel head coil at UF and an 8-channel head coil at UAB). T1-weighted three-dimensional magnetization-prepared rapid acquisition gradient-echo (MP-RAGE) images were acquired and used for analyses (TR: 7.0 ms, TE: 3.2 ms, flip angle: 8°, 1 mm iso voxels, FOV: 240 × 240 × 176, sagittal acquisition).

##### MRI processing

MP-RAGE files were processed using FreeSurfer 6.0^48^. FreeSurfer is a set of software tools for the study of cortical and subcortical anatomy^49–51^. Segmentation of subcortical and related structures (including hippocampus, amygdala, and thalamus) was performed. The cerebral cortex was parcellated into units with respect to gyral and sulcal structure^52–54^. Procedures for the measurement of cortical thickness have been validated against histological analysis^55^ and manual measurements^54,56^. FreeSurfer morphometric procedures have been demonstrated to show good test–retest reliability across scanner manufacturers and across field strengths^57,58^. MRI data were assessed for quality and participants were excluded for missing or insufficient quality data.

##### Brain structure

Participants reported knee pain consistent with or at risk for knee osteoarthritis, and also reported pain in other body sites (Mean = 6, Range 0–28 additional sites). As such, analyses were guided based on previously identified brain areas in a systematic review for musculoskeletal pain^28^ and other musculoskeletal and chronic pain research^27,29,31^. The final areas were selected a priori by a team consensus and align with our other work^30^. Mean thickness values for each cortical region (Desikan-Killiany-Tourville parcellation) and subcortical volumes were exported for statistical analyses. Metrics were the bilateral mean thickness for the rostral and caudal anterior cingulate cortices (ACC), insula, medial prefrontal cortex (MPFC), primary somatosensory cortex (S1), dorsolateral prefrontal cortex (DLPFC), and thalamus, amygdala and hippocampus volumes adjusted for total intracranial volume.

#### Environmental and sociocultural measures

The environmental and sociocultural measures used were selected based on identification in the NIA and NIMHD Frameworks under the environmental and sociocultural level of analysis/domains of influence and available in the UPLOAD2 study. Environmental and sociocultural measures included self-reported educational level, current income, number of people living in the household, employment status, current insurance status, perceived social support and experiences of interpersonal discrimination.

*Educational level (n* = *147)*—Participants reported their attained level of education according to six categories: 1 = “some school but did not complete high school,” 2 = “high school degree,” 3 = “2-year college degree,” 4 = “4-year college degree,” 5 = “master's degree,”, and 6 = “doctoral degree.”

*Income level (n* = *144)*—Annual household income was assessed in increments of $9999, starting at 1 = “$0–$9999” and continuing until the last category: 10 = “$150,000 or higher.”

*Household size (n* = *144)*—Participants were asked “Including you, how many people are living or staying at your home address?”.

*Employment status (n* = *147)*—Employment status was assessed using seven categories: “working now,” “only temporarily laid off, on sick leave or maternity leave”, “looking for work,” “unemployed”, “retired”, and “disabled, permanently or temporarily”, “student,” and “other.” Categories were then dichotomized into 1 = “working now” or “retired” or 0 = “only temporarily laid off, on sick leave or maternity leave”, “looking for work,” “unemployed”, “disabled, permanently or temporarily”, “student,” and “other”.

*Insurance Status (n* = *147)–* Participants were asked “Are you covered by health insurance or some other kind of health care plan?” as either 1 = yes or 0 = no. Participants who reported “unsure” were counted as missing.

*The Multidimensional Scale of Perceived Social Support (MSPSS)*^59^* (n* = *136)*—The MSPSS measures the perceived social support from family, friends and significant other using a 7-point Likert scale (1 = “very strongly disagree” to 7 = “very strongly agree”). Total scores are calculated as a summation of all questions with higher scores indicating greater perceived social support.

*Experiences of Discrimination (EOD) questionnaire*^60,61^* (n* = *145)*—The EOD measures incidences of self-reported experiences of interpersonal discrimination over an individual’s lifetime, as well as the frequency of each event, worry for each event, the reason certain events occurred, and response to certain situations on a 0 = “never”, 1 = “once, 2.5 = “2 to 3 times”, and 5 = “4 or more times” scale. These values are summed with higher scores signifying greater experiences of interpersonal discrimination over an individual’s lifetime.

### Statistical analysis

All data were analyzed using SAS v.9.4 (Cary, NC) and SPSS 26.0 (IBM, Chicago, IL), and checked for normality, outliers, and missing values. Differences between participant characteristics by sociodemographic groups (self-reported as NHB or NHW) were analyzed using T-Test for continuous variables and Chi-Squared or Fisher Test where appropriate for categorical variables. A total of 147 participants completed brain imaging. Income (n = 3) and household number (n = 3) were imputed from data at a second time point. Individuals missing two or fewer questions on the perceived social support had their scores imputed by using the within average of individual questions (n = 8). For individuals with three questions or more missing, perceived social support (n = 2) and discrimination (n = 1) was imputed from data at a second time point. Two participants were missing data for perceived social support or discrimination and were excluded from analysis for a final sample size of 145. A sensitivity test repeating all analyses was completed excluding individuals with imputed data to confirm findings (n = 129). Consistent with findings from our previous studies, primary explanatory variables in the model included: age, self-identified sex (1 = male, 2 = female), study site (1 = UF or 2 = UAB to account for possible scanner differences), waist circumference and total pain sites. Outcome measures for the brain ROIs: ACC, insula, MPFC, S1, DLPFC thickness, and thalamus, amygdala, and hippocampus volume. Nested linear regression modeling was completed as follows: model 1) the primary explanatory variables, including age, sex, study site, waist circumference and total pain sites; model 2) primary explanatory variables from model 1 plus environmental and sociocultural variables including education, income, household number, employment, insurance status, social support and discrimination; model 3) all variables from model 2 plus ethnic-race groups who significantly differed on additional sociodemographic factors thus identified as sociodemographic groups, NHB adults (younger with lower levels of income and education) = 1 compared to and NHW adults (older with higher income/education) = 2.

### Ethical approval

This study was conducted in accordance with the Declaration of Helsinki. The UPLOAD-2 study was approved by the University of Florida Institution Review Board (IRB approval number 201400209) on June 6, 2014, and the University of Alabama at Birmingham Institution Review Board (IRB approval number 40915002) on November 11, 2014. All participants provided verbal and written informed consent prior to any study procedures being conducted.

## Results

### Participant characteristics

Participant characteristics are displayed in Table 1. NHB adults were significantly younger with lower education and income compared to the NHW adults. As each ethnic-race group was limited in representation, the groups differed from each other on relevant sociodemographic variables, and statistical test are not able to correct group imbalances, ethnic-race group interpretations require caution and will be framed from an intersectional perspective^62,63^. Additionally, in line with an intersectional approach, the term ‘sociodemographic groups’ is used to classify the NHB and NHW groups because they differ on multiple sociodemographic factors.

**Table 1:** Sample characteristics.

Characteristics	Total	Non-Hispanic black	Non-hispanic white	*p*-value
	N = 145	N = 71	N = 74	
Primary explanatory variables				
Age, mean ± std	58.3 ± 8.0	56.2 ± 6.3	60.2 ± 9.0	0.0020
Sex, n (%)				0.2204
Male	52 (35.9)	29 (40.9)	23 (30.7)	
Female	93 (64.1)	42 (59.1)	52 (69.3)	
Waist circumference, mean ± std	102.4 ± 14.3	102.2 ± 13.4	102.6 ± 15.2	0.8576
Study site, n (%)				0.2930
University of Florida	93 (63.3)	42 (59.2)	50 (67.6)	
University of Alabama at Birmingham	54 (36.7)	29 (40.8)	24 (32.4)	
Number of pain sites, median [std]	6.0 [4.0]	5.8 [3.7]	5.5 [3.6]	0.5989
Environmental and sociocultural factors				
Annual household income, n (%)				< 0.0001
$0–$9999	34 (23.5)	24 (33.8)	10 (13.5)	
$10,000–$19,999	17 (11.7)	11 (15.5)	6 (8.1)	
$20,000–$29,999	20 (13.8)	12 (16.9)	8 (10.8)	
$30,000–$39,999	6 (4.1)	4 (5.7)	2 (2.7)	
$40,000–$49,999	12 (8.3)	3 (4.3)	9 (12.2)	
$50,000–$59,999	16 (11.0)	5 (7.0)	11 (14.8)	
$60,000–$79,999	13 (9.0)	4 (5.6)	9 (12.2)	
$80,000–$99,999	11 (7.6)	5 (7.0)	6 (8.1)	
$100,000–$149,999	11 (7.6)	2 (2.8)	9 (12.2)	
$150,000 or higher	5 (3.4)	1 (1.4)	4 (5.4)	
Education, n (%)				< 0.0001
Some school but did not complete high school	10 (6.9)	8 (11.3)	2 (2.7)	
High school degree	55 (38.0)	32 (45.1)	23 (31.1)	
2-year college degree	25 (17.2)	12 (16.9)	13 (17.5)	
4-year college degree	30 (20.7)	10 (14.1)	20 (27.0)	
Master’s degree	18 (12.4)	7 (9.8)	11 (14.9)	
Doctoral degree	7 (4.8)	2 (2.8)	5 (6.8)	
Employment status, n (%)				0.1056
Currently working/retired	100 (69.0)	44 (62.0)	56 (75.7)	
Laid off/on leave/unemployed; looking/disabled/ student	45 (31.0)	27 (38.0)	18 (24.3)	
Number in household, mean ± std	2.2 ± 1.2	2.3 ± 1.4	2.1 ± 1.0	0.4312
Current Insurance, n (%)	125 (86.2)	60 (84.5)	65 (87.8)	0.6340
Multidimensional scale of perceived social support	65.3 ± 17.6	64.5 ± 19.6	66.0 ± 15.6	0.6107
Lifetime discrimination	7.7 ± 9.4	12.5 ± 9.3	3.1 ± 7.0	< 0.0001

Between group differences were established using independent samples T-Test (two-tailed), Chi-Sq or Fisher’s Exact Test where appropriate.

### Associations between environmental and sociocultural factors and pain-related brain regions

Nested linear regression models for ROIs are displayed in Table 2.

**Table 2:** Nested linear regression analyses examining environmental and sociocultural factors in relation to brain regions associated with pain processing.

	ACC thickness	Insula thickness	MPFC thickness	S1 thickness	DLPFC thickness	Thalamus volume	Amygdala volume	Hippocampus volume
Independent variables								
Model 1: primary								
Age	− 0.3402***	− 0.2072*	− 0.1933	− 0.1851*	− 0.2469**	− 0.4359***	− 0.3445***	− 0.3009***
Sex	0.1787*	0.1319	0.0522	0.1004	0.0766	0.3347***	0.3830***	0.4464***
Study site	0.1468	0.1700*	0.0710	− 0.2334**	0.0099	− 0.0153	0.1104	0.0532
Waist circumference	− 0.1125	− 0.2287**	− 0.0402	0.0041	− 0.2057*	0.0158	− 0.0241	0.0684
Total pain sites	− 0.0325	− 0.0020	− 0.1412	− 0.0745	− 0.0590	0.0478	0.0460	0.0955
*Adj. R*^*2*^	0.1589	0.1020	−	0.0576	0.0703	0.3240	0.2969	0.3388
Model 2: Env/Soc								
Age	− 0.2435**	− 0.2459**	− 0.1631	− 0.2272*	− 0.2526**	− 0.4191***	− 0.3618***	− 0.2761***
Sex	0.1869*	0.0794	0.0380	0.0379	0.0502	0.3235***	0.3534***	0.4406***
Study site	0.1608*	0.1829*	0.0757	− 0.2152*	− 0.0056	− 0.0300	0.0897	0.0326
Waist circumference	− 0.0983	− 0.2309**	− 0.0267	− 0.0005	− 0.2012*	0.0339	− 0.0095	0.0802
Total pain sites	− 0.0030	0.0606	− 0.1068	− 0.0106	− 0.0453	0.0615	0.0401	0.0917
Education	− 0.0563	0.1109	0.0151	− 0.0340	− 0.0538	− 0.0582	− 0.1097	− 0.1105
Income	0.1431	0.2320*	0.1472	0.3417**	0.2580*	0.0446	0.1208	0.1103
Household size	0.1284	0.0712	0.0980	− 0.0053	− 0.0471	− 0.0647	− 0.0696	− 0.0991
Employment	− 0.0837	0.0019	− 0.1461	− 0.0227	− 0.0698	0.1376	0.1239	0.1239
Insurance	− 0.0955	0.0048	0.0112	− 0.1251	− 0.0102	− 0.0687	0.0373	− 0.079
Perceived social support	− 0.0234	− 0.1588	0.0572	− 0.0287	− 0.0300	0.0852	0.0079	0.0001
Discrimination	0.1100	0.0288	0.0244	− 0.1173	0.1082	0.1911*	0.1822*	0.2325**
*Adj. R*^*2*^	0.1598	0.1616	−	0.1147	0.0650	0.3419	0.3077	0.3679
*ΔR*^*2*^	0.0009	0.0596	−	0.0571	− 0.0053	0.0179	0.0108	0.0291
Model 3: sociodemographic group								
Age	− 0.2614**	− 0.2894**	− 0.1823	− 0.2845**	− 0.2582	− 0.3827***	− 0.3299***	− 0.2358**
Sex	0.1777*	0.0569	0.0281	0.0082	0.0473	0.3423***	0.3699***	0.4615***
Study site	0.1645*	0.1920*	0.0797	− 0.2033**	− 0.0044	− 0.0376	0.0830	0.0242
Waist circumference	− 0.1016	− 0.2390**	− 0.0303	− 0.0112	− 0.2023	0.0407	− 0.0036	0.0877
Total pain sites	− 0.0096	0.0445	− 0.1139	− 0.0318	− 0.0474	0.0749	0.0519	0.1066
Education	− 0.0772	0.0600	− 0.0073	− 0.1012	− 0.0604	− 0.0155	− 0.0723	− 0.0633
Income	0.1054	0.1400	0.1067	0.2205*	0.2459	0.1217	0.1883	0.1957*
Household size	0.1335	0.0838	0.1035	0.0113	− 0.0455	− 0.0752	− 0.0789	− 0.1108
Employment	− 0.0639	0.0502	− 0.1248	0.0411	− 0.0635	0.0971	0.0884	0.0790
Insurance	− 0.0769	0.0501	0.0311	− 0.0655	− 0.0042	− 0.1066	0.0041	− 0.1209
Perceived social support	− 0.0192	− 0.1485	0.0617	− 0.0151	− 0.0287	0.0766	0.0004	− 0.0094
Discrimination	0.1747	0.1868*	0.0941	0.0909	0.1289	0.0587	0.0663	0.0859
Sociodemographic Group	0.1373	0.3352***	0.1478	0.4416***	0.0438	− 0.2808***	− 0.2460**	− 0.3110***
*Adj. R*^*2*^	0.1662	0.2313	–	0.2401	–	0.3903	0.3435	0.4287
*ΔR*^*2*^	0.0064	0.0697	–	0.1254	–	0.0484	0.0358	0.0608

Standardized (beta) values reported. n = 145. ACC = anterior cingulate cortices; MPFC = medial prefrontal cortex; S1 = thalamus and the bilateral primary somatosensory cortex; DLPFC = dorsolateral prefrontal cortex.

*p < .05, **p < .01, ***p < .001.

Models 1–3 were not significant for MPFC, and Models 3 were not significant for DLPFC.

#### Rostral and caudal anterior cingulate cortices (rACC and cACC) thickness

With all variables included, the overall model explained 17% of the variance in ACC thickness. Primary explanatory variables in model 1 accounted for 16% of the variance (*F* (5, 139) = 6.44, *p* < 0.0001). Environmental and sociocultural variables entered in model 2, explained < 1% of the variance in ACC thickness (*F* (12, 132) = 3.28, *p* = 0.0004). Sociodemographic groups entered in model 3 explained < 1% of the variance (*F* (13, 131) = 3.21, *p* = 0.0003). In the final model, younger age (*beta* = -0.261, *p* = 0.0052), female sex (*beta* = 0.178, *p* = 0.0309), and UAB study site (*beta* = 0.164, *p* = 0.0419) remained significantly associated with greater ACC thickness. In a sensitivity analysis excluding those with *imputed* variables, findings were similar with the addition of lifetime discrimination (*beta* = 0.213, *p* = 0.023) indicated as significant while study site was no longer significant.

#### Insula

With all variables included, the overall model explained 23% of the variance in insula thickness. Primary explanatory variables in model 1 accounted for 10% of the variance (*F* (5, 139) = 4.27, *p* = 0.0012). Environmental and sociocultural variables entered in model 2, explained 6% of the variance in insula thickness (*F* (12, 132) = 3.31, *p* = 0.0003). Sociodemographic groups entered in model 3 explained 7% of the variance (*F* (13, 131) = 4.33, *p* < 0.0001). In the final model, younger age (*beta* = -0.289, *p* = 0.0013), UAB study site (*beta* = 0.192, *p* = 0.0138), smaller waist circumference (*beta* = -0.239, *p* = 0.0019), greater lifetime discrimination (*beta* = 0.187, *p* = 0.0420), and the NHW sociodemographic group (*beta* = 0.335, *p* = 0.0004) were significantly associated with greater insula thickness. In a sensitivity analysis excluding those with imputed variables, findings remained the same with the addition that perceived social support (*beta* = -0.175, *p* = 0.040) also showed significance.

#### Medial prefrontal cortex (MPFC) thickness

The overall models were not statistically significant (*p* = 0.1304, *p* = 0.3431, *p* = 0.2858). In a sensitivity analysis excluding those with imputed variables, models were not significant (*p* = 0.233, *p* = 0.454, *p* = 0.368).

#### Bilateral primary somatosensory Cortex (S1) thickness

With all variables included, the overall model explained 24% of the variance in bilateral S1 thickness. Primary explanatory variables in model 1 accounted for 6% of the variance (*F* (5, 139) = 2.76, *p* = 0.0208). Environmental and sociocultural variables entered in model 2, explained 6% of the variance in bilateral S1 thickness (*F* (12, 132) = 2.56, *p* = 0.0045). Sociodemographic groups entered in model 3 explained 13% of the variance (*F* (13, 131) = 4.50, *p* < 0.0001). In the final model, younger age (*beta* = − 0.285, *p* = *0.0*015), UF study site (*beta* = − 0.203, *p* = *0.0*088), greater income (*beta* = 0.220, *p* = 0.0491) and the NHW sociodemographic group (*beta* = 0.442, *p* < 0.0001) remained significantly associated with greater S1 thickness. In a sensitivity analysis excluding those with imputed variables, findings were similar, however, income was no longer significant.

#### Dorsolateral prefrontal cortex (DLPFC) thickness

Primary explanatory variables in model 1 accounted for 7% of the variance (*F* (5, 139) = 3.18, *p* = 0.0095). Model 2 (*F* (12, 132) = 1.83, *p* = *0.0*487) and model 3 (*F* (13, 131) = 1.70, *p* = *0.0*686) did not account for any additional variance. In a sensitivity analysis excluding those with imputed variables, no differences were identified.

#### Thalamus volume

With all variables included, the overall model explained 39% of the variance in thalamus volume. Primary explanatory variables in model 1 accounted for 32% of the variance (*F* (5, 139) = 14.81, *p* < 0.0001). Environmental and sociocultural variables entered in model 2 explained 2% of the variance in thalamus volume (*F* (12, 132) = 7.24, *p* < 0.0001). Sociodemographic groups entered in model 3 explained 5% of the variance (*F* (13, 131) = 8.09, *p* < 0.0001). In the final model, younger age (*beta* = − 0.383, *p* < 0.0001), female sex (*beta* = 0.342, *p* < 0.0001) and the NHB sociodemographic group (*beta* = − 0.280, *p* = 0.0009) remained significantly associated with greater thalamus volume. In a sensitivity analysis excluding those with imputed variables, significant variables remained the same.

#### Amygdala volume

With all variables included, the overall model explained 34% of the variance in amygdala volume. Primary explanatory variables in model 1 accounted for 30% of the variance (*F* (5, 139) = 13.16, *p* < 0.0001). Environmental and sociocultural variables entered in model 2, explained 1% of the variance in amygdala volume (*F* (12, 132) = 6.33, *p* < 0.0001). Sociodemographic groups entered in model 3, explained 3% of the variance (*F* (13, 131) = 6.80, *p* < 0.0001). In the final model, younger age (*beta* = − 0.330, *p* < 0.0001), female sex (*beta* = 0.370, *p* < 0.0001), and the NHB sociodemographic group (*beta* = − 0.246, *p* = 0.0049) remained significantly associated with greater amygdala volume. In a sensitivity analysis excluding those with imputed variables, significant variables remained with income (*beta* = 0.216, *p* = 0.041) also indicated.

#### Hippocampus volume

With all variables included, the overall model explained 43% of the variance in hippocampus volume. Primary explanatory variables in model 1 accounted for 34% of the variance (*F* (5, 139) = 15.75, *p* < 0.0001). Environmental and sociocultural variables entered in model 2, explained 3% of the variance in hippocampus volume (*F* (12, 132) = 7.99, *p* < 0.0001). Sociodemographic groups entered in model 3, explained 6% of the variance (*F* (13, 131) = 9.31, *p* < 0.0001). In the final model, younger age (*beta* = − 0.236, *p* = 0.0024), female sex (*beta* = 0.461, *p* < 0.0001), greater income (*beta* = 0.196, *p* = 0.0441), and the NHB sociodemographic group (*beta* = − 0.311, *p* = 0.0002) remained significantly associated with hippocampus volume. In a sensitivity analysis excluding those with imputed variables, significant variables remained with lifetime discrimination (*beta* = 0.174, *p* = 0.028) also showing significance.

## Discussion

Guided by the NIA and NIMHD Health Disparities Research Frameworks^2,38,39^, the current study aimed to identify the contributions of environmental and sociocultural factors on pain-related brain structures in a sociodemographically diverse group of adults reporting knee pain^41^. As hypothesized, environmental and sociocultural factors were associated with pain-related brain structures. Ethnicity-race remained a small but significant predictor across several models. It is important to note that our study included only a few of the extensive array of environmental and sociocultural factors contributing to health disparities warranting investigation^2^. Findings are presented in alignment with the National Institutes of Health reporting requirements for ethnicity and race, which can provide consistent terminology for comparisons across studies^2,64^. Despite these efforts, significant heterogeneity remains within self-identified ethnicity and race categories. We also incorporate an intersectional approach by providing more specific sociodemographic information for each group which promotes more accurate interpretations and will better inform efforts to improve health for all^63^.

### Environmental and sociocultural contributions to structural differences in brain regions associated with pain processing

Associations between experiences of chronic pain and alterations in brain morphology are well-established^28,65,66^. Our previous publications in the same study sample indicated greater gray matter across cortical and subcortical areas of the brain in the early stages of chronic MSK pain and lesser gray matter with persisting, high stage chronic pain^40,41^. Self-identified ethnicity-race was also identified as a significant predictor. Our previous research in the temporal lobe regions of the brain show socioenvironmental factors explained the sociodemographic group differences observed^36,40^. Our current findings in recognized pain-related regions of the brain are similar. Inclusion of available factors from the NIA and NIMHD Frameworks specific to the environmental and sociocultural levels of analysis/domains of influence help explain the variance observed in pain-related brain structures. Brain function and structure are highly influenced by life experiences^32–35^. By incorporating available and recognized environmental and sociocultural variables in study models, we begin the process of systematically disentangling and identifying the factors contributing to health-related outcomes at the neurobiological level^2,67,68^.

Not surprisingly, age was the strongest and most consistent predictor in all of the models. Age-related changes in pain and brain structure are well recognized^40,41^. Sex differences in brain structures are also well established^30^. Less commonly considered is the cumulative impact of environmental and sociocultural experiences^36^. *Our findings show that considering demographic factors (age, sex, ethnicity-race) alone is not sufficient in brain imaging analyses, inclusion of key socioenvironmental factors is also necessary. Further, to advance health disparities research and improve health for all, the heterogeneous classification of ethnicity and race requires additional “intersectional detail” regarding specific sociodemographic descriptions of the self-identified ethnicity-race groups represented.*

### Strengths, limitations, and future directions

The study benefitted from a large and ethnically diverse sample with data collected from two study sites (Gainesville, Florida and Birmingham, Alabama). Validated instruments and standardized procedures were used where applicable. With brain imaging data on 147 individuals, the sample size extends beyond typical pain and imaging analyses. Additionally, the sociodemographic diversity within our self-reported ethnic-race groups highlights the importance of reporting within group differences to contribute to a more informative “intersectional” understanding of the study samples represented^63^.

There are limitations to acknowledge, as the study is cross-sectional, longitudinal data will improve understanding of the relationships between environmental and sociocultural factors, pain, and pain-related brain structures. Further, participants in the study had knee pain with or at risk for knee OA with many reporting chronic pain at other body sites. They do not represent individuals with more severe knee OA nor those with primary chronic musculoskeletal pain at other body sites. Additionally, a few of the measures capturing environmental and sociocultural variables were categorical in nature and may not optimally capture the constructs of interest. Additionally, further investigations are needed on the extensive array of factors across different levels of analysis associated with health disparities. Despite limitations in study design and measures, findings provide an important foundation for improving the understanding of the combined influence of environmental, sociocultural, demographic, and pain-related factors on pain-related brain structures.

## Conclusions

Disparities in pain-related experiences are well-established. A growing body of evidence indicates the role of environmental and sociocultural factors in contributing to these observed differences. The contributions of environmental and sociocultural factors on pain-related brain structures have been minimally investigated. Our findings show that with inclusion of environmental and sociocultural factors, e.g., education, income, household number, employment, insurance status, social support, and discrimination; a significant proportion of variance within pain-related brain structures is explained. Further investigations of the vast array of additional environmental and sociocultural variables are needed to continue the processes of disentangling and identifying the factors contributing to disparities in health outcomes. Additionally, our study included a balanced representation of NHB and NHW adults. Despite this strength, significant heterogeneity remains even with a combined classification of ethnicity and race^63^. Consistent with an intersectionality theoretical framework, we provide additional sociodemographic descriptions of the self-identified ethnic-race groups represented in our study to improve interpretations and inform research efforts moving forward.

## Data Availability

Data is not publicly available but can be requested from the corresponding author.
